# Development and validation of a high density SNP genotyping array for Atlantic salmon (*Salmo salar*)

**DOI:** 10.1186/1471-2164-15-90

**Published:** 2014-02-06

**Authors:** Ross D Houston, John B Taggart, Timothé Cézard, Michaël Bekaert, Natalie R Lowe, Alison Downing, Richard Talbot, Stephen C Bishop, Alan L Archibald, James E Bron, David J Penman, Alessandro Davassi, Fiona Brew, Alan E Tinch, Karim Gharbi, Alastair Hamilton

**Affiliations:** 1 The Roslin Institute and Royal (Dick) School of Veterinary Studies, University of Edinburgh, Midlothian EH25 9RG, UK; 2 Institute of Aquaculture, University of Stirling, Stirling FK9 4LA, UK; 3 Edinburgh Genomics, Ashworth Laboratories, King’s Buildings, University of Edinburgh, Edinburgh, EH9 3JT, UK; 4 Edinburgh Genomics, The Roslin Institute, University of Edinburgh, Midlothian EH25 9RG, UK; 5 Affymetrix UK Ltd, Voyager, Mercury Park, Wycombe Lane, Wooburn Green, High Wycombe HP10 0HH, UK; 6 Landcatch Natural Selection Ltd., 15 Beta Centre, Stirling University Innovation Park, Stirling FK9 4NF, UK

**Keywords:** Atlantic salmon, *Salmo salar*, Polymorphism, Single nucleotide polymorphism, SNP, Next-generation sequencing, Array, Genomics, Mapping, Genome duplication

## Abstract

**Background:**

Dense single nucleotide polymorphism (SNP) genotyping arrays provide extensive information on polymorphic variation across the genome of species of interest. Such information can be used in studies of the genetic architecture of quantitative traits and to improve the accuracy of selection in breeding programs. In Atlantic salmon (*Salmo salar*), these goals are currently hampered by the lack of a high-density SNP genotyping platform. Therefore, the aim of the study was to develop and test a dense Atlantic salmon SNP array.

**Results:**

SNP discovery was performed using extensive deep sequencing of Reduced Representation (RR-Seq), Restriction site-Associated DNA (RAD-Seq) and mRNA (RNA-Seq) libraries derived from farmed and wild Atlantic salmon samples (n = 283) resulting in the discovery of > 400 K putative SNPs. An Affymetrix Axiom® myDesign Custom Array was created and tested on samples of animals of wild and farmed origin (n = 96) revealing a total of 132,033 polymorphic SNPs with high call rate, good cluster separation on the array and stable Mendelian inheritance in our sample. At least 38% of these SNPs are from transcribed genomic regions and therefore more likely to include functional variants. Linkage analysis utilising the lack of male recombination in salmonids allowed the mapping of 40,214 SNPs distributed across all 29 pairs of chromosomes, highlighting the extensive genome-wide coverage of the SNPs. An identity-by-state clustering analysis revealed that the array can clearly distinguish between fish of different origins, within and between farmed and wild populations. Finally, Y-chromosome-specific probes included on the array provide an accurate molecular genetic test for sex.

**Conclusions:**

This manuscript describes the first high-density SNP genotyping array for Atlantic salmon. This array will be publicly available and is likely to be used as a platform for high-resolution genetics research into traits of evolutionary and economic importance in salmonids and in aquaculture breeding programs via genomic selection.

## Background

Atlantic salmon (*Salmo salar*) is a species of great economic, environmental and scientific importance, with a worldwide production of approximately 1.4 million tonnes per annum [1]. Atlantic salmon is also considered a model species for the other members of the Salmonidae family and as such is the target of an on-going genome sequencing and assembly project [2]. This genome sequence and its interrogation will be important for understanding the genetic regulation of complex traits in salmonids, with applications for improvement of aquaculture breeding programs and for population and evolutionary genetics studies. However, unlike major terrestrial farmed species, a high-throughput high-density genotyping array is not yet available for screening genome-wide polymorphic variation in Atlantic salmon. An existing low-density single nucleotide polymorphism (SNP) array contains approximately 6 K polymorphic SNPs [3].

The genetic improvement of Atlantic salmon through selective breeding programs began in the early 1970s in Norway [4] and, despite a 3 - 4 year generation interval, has resulted in rapid improvement of economically-important traits such as growth, sexual maturation and disease resistance [5]. Microsatellite and SNP marker resources have been developed and applied in breeding programs for parentage assignment [6] and quantitative trait loci (QTL) detection with subsequent marker-assisted selection for favourable alleles, particularly for increased disease resistance (*e.g.*[7-9]). SNPs are increasingly applied as the marker of choice for genetic studies due to their abundance, ease of discovery and low cost of genotyping per locus, especially using SNP chips which simultaneously assay tens of thousands of SNPs per sample. Genotyping-by-sequencing approaches such as Restriction Site-associated DNA (RAD) sequencing [10] are increasingly utilised to simultaneously discover and genotype thousands of SNPs in salmonid species with applications for genome characterisation, population genomics and QTL mapping [11-13]. Additionally, the existing 6 K SNP array [3] has been applied for mapping QTL [14,15] and differentiating between populations [16,17].

The SNP density offered by either the existing SNP array or RAD sequencing approaches to date is not sufficient to capture population-wide linkage disequilibrium to enable fully effective genome-wide association studies (GWAS) [18]. Further, dense genome-wide SNP data can also be included in breeding programs alongside extensive phenotype and pedigree information to increase the accuracy of selection for key traits using genomic selection [19,20]. Genomic selection has the potential to dramatically increase selection accuracy, genetic gain and reduce inbreeding in Atlantic salmon breeding programs [21]. Genotyping tools used for GWAS and genomic selection in terrestrial species with genomes of comparable size to Atlantic salmon contain between ~50 K to ~800 K SNPs [22-26], highlighting the need for a denser Atlantic salmon genotyping platform.

Salmonids such as Atlantic salmon are descended from a teleost lineage which has undergone a whole genome duplication event approximately 25 - 100 million years ago and are thought to be in the process of reverting to a diploid state [27,28]. This genome duplication complicates the discovery of genuine bi-allelic SNPs as it can be difficult in bioinformatics analyses to distinguish variation between paralogous loci from genuine SNP variation at unique genome locations (*e.g.*[12,29-31]). High-throughput sequencing technologies now make large scale SNP discovery in salmonids attainable (e.g. [3,11-13,30-32]), subject to high sequence coverage of both alleles. Full genome re-sequencing for salmonid SNP discovery remains expensive and genome complexity reduction techniques such as reduced-representation sequencing (RR-Seq), RAD sequencing (RAD-Seq) and RNA sequencing (RNA-Seq) have all been successfully applied for this purpose (*e.g.*[30-33]).

The aim of the current study was to develop a high-density SNP genotyping array for Atlantic salmon and to validate these SNPs and the array by genotyping samples from several populations of farmed and wild fish. Due to the complexities of the Atlantic salmon genome, a multi-faceted approach to SNP discovery was applied using a combination of RR-Seq, RAD-Seq and RNA-Seq alongside several strategies for exclusion of paralogous sequence variants (PSV) including RR-Seq of haploid material. This manuscript describes the creation and testing of the SNP array and highlights its potential applications in Atlantic salmon genetics research.

## Results and discussion

### Sequencing and SNP discovery

To generate candidate SNPs for inclusion on an Affymetrix Axiom® myDesign Custom Array (named ‘ssalar01’,) three main Illumina – based sequencing strategies were applied; RR-Seq (56 fish), RAD-Seq (160 fish) and RNA-Seq (72 fish). SNPs were discovered in representative samples of the Atlantic salmon breeding company Landcatch Natural Selection Ltd (LNS, Stirling, UK) while the RR-Seq also included a pool of wild fish from diverse geographical sources and a single haploid Atlantic salmon embryo for the purpose of PSV exclusion (See Methods and Table 1 for details).

**Table 1:** Summary of the sequencing experiments for SNP discovery

	**RR-Seq**	**RAD-Seq**	**RNA-Seq**
Samples (number)	Farmed (40), Wild (16), Haploid (1)	Farmed (160)	Farmed (72)
Sequencing	Illumina 100 bp PE	Illumina 100 bp S&PE	Illumina 100 bp PE
Initial putative SNPs	472,072	467,268	816,570
SNPs for array design	99,097	83,151	229,754
Final SNPs on array	73,800	54,197	156,979

### SNP selection and filtering

Alignment of the Illumina sequence data to the draft Atlantic salmon reference genome assembly (NCBI Assembly GCA_000233375.1) identified 472,072 (RR-Seq), 467,268 (RAD-Seq) and 816,570 (RNA-Seq) putative variable SNP positions. Following the quality-control filtering of these putative SNPs (described in ‘Methods’), 99,097 (RR-Seq), 83,151 (RAD-Seq) and 229,754 (RNA-Seq) candidate SNPs remained for potential inclusion on the ‘ssalar01’ array. In addition to the newly-discovered candidate SNPs, a number of predominantly public domain, mapped SNPs (n = 4880) were also included. All candidate SNPs (total of 411,308; 4,139 of which were detected in more than one SNP discovery category) were submitted to Affymetrix for *in silico* prediction of their probability of conversion to a reliable assay on the Axiom array (p-convert score). Following application of filtering criteria incorporating the p-convert score (see Methods) the final array contained 286,021 putative SNPs assayed by 443,627 probes. Of the SNP on the array, 3,369 were detected in more than one of the SNP discovery categories (Additional file 1: Table S1).

### Performance of SNPs on the array

The ssalar01 array was used to genotype 96 Atlantic salmon samples of diverse origin from three main categories; farmed Scottish (four year-groups of the Landcatch Natural Selection broodstock population; n = 47); farmed Norwegian (two groups of samples derived from two major breeding companies; n = 16); and wild fish (sourced from Scotland, Ireland, Norway and Spain; n = 33) (see Methods and Additional file 2). Following assessment of the cluster properties of each of the SNPs, a total of 135,682 SNPs were designated as high quality and polymorphic. The main reason for discarding SNPs at this stage was the high rate of monomorphism, with 110,910 SNPs designated as monomorphic in these 96 DNA samples (Table 2). Given that the samples contained either the same or closely-related samples to the SNP discovery populations, it is unlikely that there would be a failure to observe the minor allele of a genuine SNP. Following a final filtering stage based on exclusion of any genotyped SNPs showing an apparent Mendelian error in the pedigreed samples, the final set of 132,033 QC-filtered SNPs were used for further analysis (Table 2).

**Table 2:** Quantity and source of the SNPs on the array at different stages of quality filtering

**SNP category**	**RR seq**	**RAD seq**	**RNA seq**	**Other**	Total*
Total candidate SNPs	73,800	54,197	156,979	4,714	286,021
Low quality clusters**	9,795	8,192	21,609	219	39,429
Monomorphic	9,010	18,157	83,368	811	110,910
High quality polymorphic SNPs	54,995	27,848	52,002	3,684	135,682
Mendelian error	1,292	756	1,595	66	3,649
Final total filtered SNPs	53,703	27,092	50,407	3,618	132,033

*The row total is lower than sum of the individual column totals because a proportion of these SNPs were from multiple categories (*e.g.* discovered in both the RR and RAD experiments); see Additional file 1: Table S1.

**‘Quality’ refers to the cluster properties of the SNP when genotyped on the Axiom array. Low quality SNPs are those with cluster properties that fall below a threshold value (*e.g.* < 97% call rate).

**Figure 1:**
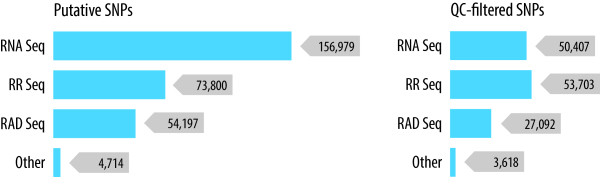
**Source of the SNPs on the ssalar01 array.** Proportion of total SNPs derived from each of the SNP discovery categories (RR-Seq, RAD-Seq, RNA-Seq and other). ‘Putative SNPs’ comprise the 286,021 putative SNPs placed on the array, and ‘QC-filtered SNPs’ comprise the 132,033 final quality-control filtered SNPs used for analysis. Note that some SNPs were detected in multiple discovery categories (see Additional file 1: Table S1).

The number of SNPs in the final QC-filtered dataset has a relatively even distribution across the three main SNP discovery techniques (Figure 1). By comparison, the majority of candidate SNPs provided to Affymetrix were derived from RNA-Seq, reflecting the high discrepancy between candidate and verified RNA-Seq-derived SNPs (68% drop out). While RNA-Seq has been successfully applied to detect QTL-associated SNPs in salmonids [30], the technique is purported to be particularly susceptible to false positive SNP discovery even in species with well-characterised reference genomes (*e.g.*[34]). Interestingly, the RAD-Seq-derived SNPs showed a much higher discrepancy between candidate and verified SNPs (50% drop out) than RR-Seq (32% drop out, Table 2). This may be due to the more effective removal of putative paralogous variants discovered via the RR-Seq of the haploid fish. It is also worth noting that 23% of the previously published SNPs were not verified in our sample, despite the fact that these SNPs have previously been verified through genotyping and linkage mapping experiments (*e.g.*[3,34]). This may partly reflect the different origins of the samples used in the current study and/or limitations in the genotyping technology.

A disparity between the number of putative DNA-sequencing-derived SNPs and the number of validated SNPs has been a feature of SNP discovery studies, particularly in salmonid species (*e.g.*[29,31,33]). One of the possible reasons for the apparently large number of false positive SNPs discovered in these sequencing experiments is the duplicated nature of the Atlantic salmon genome due to the whole genome duplication event approximately 25 to 100 million years ago [2]. Although analyses were performed to remove putative paralogous variants in the current study via exclusion of haploid-derived heterozygous putative SNPs (RR-Seq) and SNPs showing Mendelian errors in pedigreed samples (RAD-Seq), it is likely that a significant proportion would remain. This is particularly the case in the RNA-Seq dataset where these quality control measures were not possible. Several other possible reasons for false positives could include sequencing errors and unknown (and therefore unmasked) repeat elements; the Atlantic salmon genome is known to contain very frequent, long and similar repeats [35].

The duplicated genome also had to be accounted for when clustering the genotypes on the Axiom array. Probes designed to detect SNP alleles in a single genome location can often also detect paralogous alleles which gives rise to multi-site variants (MSV; [36]). MSVs therefore have four alleles rather than two and the clustering algorithm must distinguish between these categories. For example, in the case where the SNP (A/B) segregates in one paralogue and the other paralogue is fixed for A/A then the three possible bi-locus genotypes are AAAA, AAAB and AABB. These cluster patterns are evident from graphs of the clusters observed within the polymorphic high resolution category of SNPs. The Affymetrix AxiomGTv1 algorithm (a fine-tuned version of the BRLMM-P algorithm [37]) was applied to adapt pre-positioned clusters to the data using a Bayesian approach (see ‘Methods’). The adaptability of this algorithm will facilitate accurate genotyping of other populations, and potentially other salmonid species, which may have dissimilar MSV structures.

Finally, it is noteworthy that in the final set of QC-filtered SNPs, at least 38% (the RNA-Seq-derived SNPs) are from transcribed regions of Atlantic salmon genome (Figure 1) and therefore more likely to be functional, and this putative enrichment is advantageous for determining the genetic architecture of traits of economic or environmental importance and for comparative mapping between salmonids and more distantly-related species.

### Population segregation of SNPs

**Figure 2:**
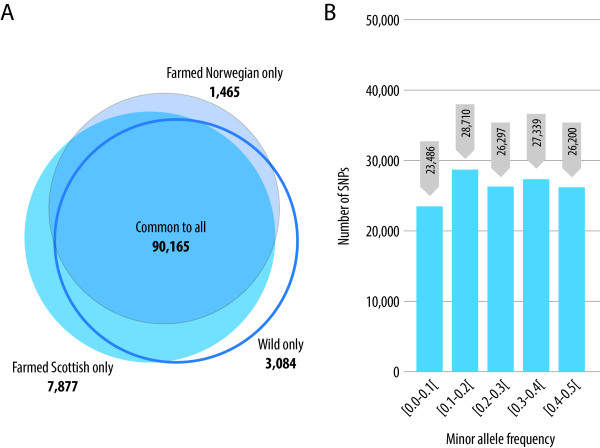
**Population segregation of SNPs and minor allele frequency. (A)** Sharing of the QC-filtered SNPs (with minor allele frequency higher than 0.05) between the different Atlantic salmon populations depicted by a Venn diagram (number of SNPs given in parentheses). **(B)** Distribution of the minor allele frequency of the final QC-filtered SNPs across all unrelated animals in the test population.

The segregation of the filtered SNPs in the samples from distinct populations of Atlantic salmon was evaluated. In the case where family samples were included, only the (unrelated) parental fish were included in the analysis (Table 3). The filtered SNPs are highly polymorphic in all three groups, with ~120 K, 102 K and 110 K SNPs had a MAF higher than 0.05 in the farmed Scottish, farmed Norwegian and wild fish respectively (Table 3). Over 90 K SNPs had a MAF over 0.05 in all three groups with only a small percentage of SNPs being polymorphic in one group only (Figure 2A). The largest number of population-specific segregating SNPs were detected in the farmed Scottish group with ~8K SNPs observed exclusively in this population. This is likely to be due to the fact that this population made up most of the SNP discovery panel for all three sequencing experiments, and that the farmed Scottish group were most highly represented in the validation population giving a higher likelihood of detecting rare minor alleles (i.e. ascertainment bias). To support this theory, the average MAF of the SNPs specific to the farmed Scottish population was 0.14; whereas the overall average MAF for the farmed Scottish population was 0.28 (only SNPs with MAF over 0.05 were included in this calculation). Across all genotyped samples and all SNPs the average MAF was 0.25, and the SNPs were evenly distributed in MAF bins ranging from 0 to 0.5 (Figure 2B).

**Table 3:** Frequency of the filtered SNPs in the tested populations (four yeargroups of farmed Scottish fish, two populations of farmed Norwegian, and a combination of the wild fish)

**Population**	Sample size*	**Number of SNPs segregating (with MAF ≠ 0)**	**Number of SNPs segregating (with MAF > 0.05)**
Overall	68	132,033	122,063
Farmed Scottish	39	130,062	120,157
Year-group 1	10	121,849	109,487
Year-group 2	10	117,182	105,752
Year-group 3	9	117,185	117,184
Year-group 4	10	117,290	105,111
Farmed Norwegian	16	108,885	101,536
Population 1	8	97,631	97,631
Population 2	8	74,154	74,154
Wild	13	119,526	110,320

*Note that only unrelated animals from each population were included; *i.e.* the offspring from trios (Farmed Scottish) or linkage mapping families (Wild) were removed. The full list of animals is given in Additional file 2.

### Genomic distribution of SNPs

**Figure 3:**
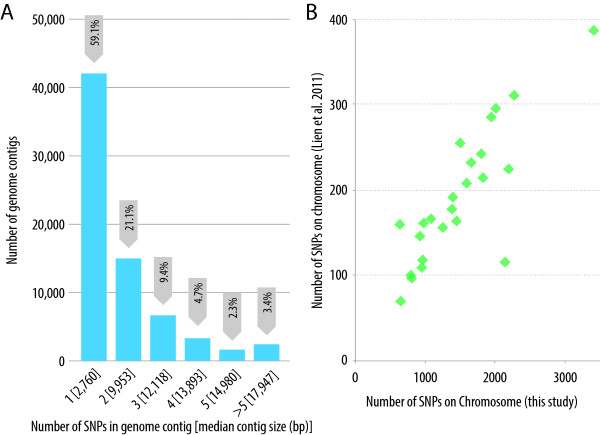
**Genomic distribution of SNPs and comparison of linkage maps. (A)** Number of final QC-filtered SNPs per reference genome contig; Number of final QC-filtered SNPs contained per reference genome contig. **(B)** Scatterplot of number of SNPs per chromosome comparing the current study to the map of Lien et al. [3]. Note that chromosomes 2, 6, 22 and 23 are not included because the number of SNPs on those chromosomes is underestimated in the current study (see ‘Methods’).

The Atlantic salmon genome is currently being sequenced and assembled [2] and the first draft publicly available assembly consists of ~2.4 gigabases of sequence data assembled into ~550,000 contigs with a contig N50 of 9.3 kb (NCBI Assembly GCA_000233375.1). The distribution of the ssalar01 array QC-filtered SNPs across the reference genome contigs was investigated. Approximately 71 K contigs contained one or more SNPs with the majority of those (59%) containing only one SNP. Only 3% of the SNP-containing contigs contained six or more SNPs (Figure 3A). The total length of the SNP-containing contigs was ~777 Mb which is approximately one third of the total assembled genome sequence. While these contigs have largely yet to be assigned to chromosomes, this indicates that the SNPs are most likely spread over a large proportion of the Atlantic salmon genome, as confirmed by the linkage mapping results given below. The average spacing between QC-filtered SNPs was ~18 kb based on the entire reference genome assembly (~2.4 gb) and ~6 kb based on the total length of the genome contigs that contained a SNP (~777 mb). As expected, the number of SNPs on a genome contig was related to the contig length with longer contigs more likely to harbour multiple SNPs (Figure 3A).

Recombination is greatly repressed in large sections of the genome of male salmonids compared to females, with large sections of the chromosomes proximal to the centromere showing close to zero recombination [28,38,39]. As a result, large haplotypes of marker alleles are inherited from sires to offspring as a single chromosomal unit [12,40]. This phenomenon was exploited to map a proportion of the sire-heterozygous SNPs in the two SalMap reference families Br5 and Br6 [41] (using 12 samples per family) to a putative linkage group and therefore chromosome. In all, 43,696 QC-filtered SNPs had the segregation pattern AB (sire) × (AA or BB) (dam) in at least one of the families. Genotypes at anchor markers from each chromosome were included (Additional file 1: Table S2) and the clustering of markers to putative linkage groups was performed using CriMap v2.4 [42] as modified by Xuelu Liu (Monsanto, USA). A total of 40,214 (92%) sire-segregating SNPs were mapped to an Atlantic salmon chromosome (Additional file 3) which, given the small size of the mapping panel, highlights the widespread lack of male recombination. All chromosomes had good SNP coverage, ranging from 589 to 3,411 SNPs per chromosome (Table 4), which is substantially higher than any published Atlantic salmon linkage map. The number of SNPs per chromosome showed a high positive correlation (r = 0.84, Figure 3B) with the SNP linkage map of Lien et al. [3] which was created by an independent group, using different SNP discovery techniques and genetic material. Therefore, it is likely that the number of SNPs discovered on each chromosome in both studies is correlated with chromosome size. These results are indicative of the validity of the chromosome assignment for the SNPs, and suggestive of their genome-wide distribution.

**Table 4:** **Number of SNPs assigned to the Atlantic salmon chromosomes using sire-based linkage mapping (chromosome and linkage group nomenclature as given in **[43]**)**

**Chromosome**	**Linkage group**	**Number of SNPs**
1	17	3,411
2	1	774
3	11	2,007
4	28	2,189
5	12	1,496
6	4	657
7	24	633
8	19	634
9	10	2,266
10	2	2,015
11	9	1,641
12	6	1,804
13	5	1,942
14	3	1,576
15	8	1,830
16	23	1,398
17	22	1,085
18	16	1,443
19	13	1,247
20	25	1,381
21	14	950
22	32	652
23	18	589
24	7	2,134
25	20	950
26	21	927
27	15	980
28	33	807
29	31	796
Total		40,214

### Identity-by-state clustering and multidimensional scaling

**Figure 4:**
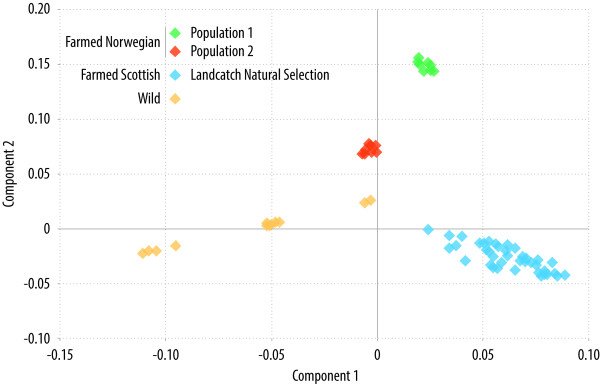
**Clustering of samples based on genetic similarity.** Clustering of samples based on genome-wide identity-by-state and multidimensional scaling to detect population structure.

Dense genome-wide SNP data can be used to estimate the overall similarity of the genomes of any two samples by calculating average measures of identity-by-state (IBS) of the marker loci. This analysis can be useful for detecting population structure in genetics studies; *e.g.* to detect and account for population stratification in GWAS or to differentiate the origin of individuals in a mixed-population sample. To evaluate the utility of the ‘ssalar01’ array to detect population structure, an *N* × *N* matrix of genome-wide IBS pairwise distances was calculated for all unrelated genotyped samples and classical multidimensional scaling of the data was applied using Plink [44]. A scatterplot of the individuals on the first two dimensions clearly reveals the clustering of samples according to their origin with distinct groups for the farmed Scottish population, the two farmed Norwegian populations and the diverse samples of wild fish (Figure 4). The number of fish included per population was relatively small, particularly for the wild samples, and genotyping additional unrelated samples from each of these (and other) populations would be advantageous to fully evaluate the utility of the array to detect population structure.

### Predicting phenotypic sex using Y-specific probes

**Figure 5:**
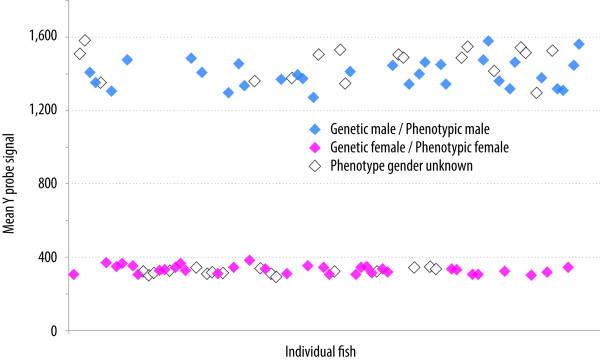
**Use of Y-specific probes to predict phenotypic sex.** Correspondence between genetic sex of the fish (based on the Y-specific probes on the array) and phenotypic sex (where known).

Apart from sexually mature individuals, identification of phenotypic sex in salmonids requires dissection of the body cavity and, in the case of juveniles, microscopic examination of gonadal tissue. The sex determining system of salmonids is primarily male heterogametic (XX/XY). A Y-specific master sex-determining gene (sdY) was recently described in rainbow trout [45], with homologues identified in other salmonid species [46]. To enable the sex of the Atlantic salmon genotyped on the SNP array to be inferred, partial sequence of the Atlantic salmon sdY gene (Additional file 4) was used to design a set of 87 putative Y specific probes (Additional file 5) while were placed on the array. The mean intensity values for these probes showed a clear clustering of the 96 genotyped samples into two groups (putative male and female) and, for 63 of the samples where phenotypic sex was known, there was a 100% concordance with the predicted sex given by the Y-specific probes (Figure 5). These results provide evidence that the same sex-determining locus acts in these Atlantic salmon populations as in rainbow trout, and that the ssalar01 array incorporates an accurate molecular genetic test for this male specific fragment, allowing robust inference of phenotypic sex in farmed and wild Atlantic salmon.

## Conclusions

This manuscript describes the creation and analysis of the first high-density (~130 K) SNP array for Atlantic salmon. The three major SNP discovery techniques (RR-Seq, RAD-Seq and RNA-Seq) all proved successful in discovering tens of thousands of high quality polymorphic SNPs in the Atlantic salmon genome. Linkage mapping and integration with the draft reference genome sequence suggests the SNPs are distributed widely over all chromosomes. This Affymetrix Axiom SNP array will be publicly available from March 2014 and will facilitate high-resolution studies to determine the genetic architecture of traits of economic and ecological importance, to study the structure of Atlantic salmon populations and to apply genomic selection in breeding programs.

## Methods

### Creation of haploid Atlantic salmon

Atlantic salmon milt (Landcatch, UK) was diluted to a concentration of 5 × 10^8^ ml^-1^ in modified Cortland’s solution, then a 2 ml aliquot was placed in a 5 cm diameter petri dish and irradiated with 254 nm UV light for 8 min at a dose rate of 170 μWcm^-2^ (optimization of irradiation protocol not shown). Irradiated milt was used to fertilize Atlantic salmon eggs (Landcatch, UK), which were then incubated under standard conditions. Putative haploids were sampled at 300 degree-days post-fertilization. Haploidy was verified by: (i) genotyping a sub-sample of these embryos (along with parents and diploid controls) using the 10 microsatellite marker multiplex system described in [47]; (ii) another sample from the same group was incubated to hatch to verify that they showed the typical “haploid syndrome” (small size and curved trunk compared to diploid controls [48]). Production of haploid embryos complied with the Animals (Scientific Procedures) Act 1986.

### Animals and preparation of sequencing libraries

#### (i) RR-Seq

Six libraries were created for RR-Seq using the restriction enzyme *Hae*III. Libraries 1 - 4 each corresponded to a pool of genomic DNA of ten fish (five male, five female) from each of the four year-group subpopulations of the LNS broodstock population. Library 5 comprised a pool of genomic DNA from 16 wild fish (sex unknown) with four from each of four populations sources in Scotland, Norway, Ireland and Spain, respectively. Library 6 comprised a single haploid fish and was sequenced for the purpose of identification and exclusion of PSV. A heterozygous base called in this single haploid individual most likely represent variation between paralogous loci (i.e. a PSV) rather than genuine SNP variation at a single unique genomic location. For libraries 1 - 5, equal amounts of individual genomic DNA was multiplexed to form pools of total 15 μg and, for library 6.5 μg genomic DNA from the haploid sample was used. These pools were subsequently digested with 15U *Hae*III (Promega, USA) for 3 hours. Genomic DNA fragments of between 450-550 bp were size-selected by agarose gel electrophoresis and the gel slices were purified using a MinElute Gel Extraction kit (Qiagen, UK). The Illumina Truseq DNA Sample Preparation Kit v2 (Illumina Inc., USA) protocol was then followed. Libraries were quantified using the Bioanalyzer 2100 (Agilent, USA), library-specific nucleotide barcodes were added, and they were sequenced in multiplexed pools on the Illumina Hiseq 2000 instrument using a 100 base paired-end sequencing strategy (v3 chemistry). All RR sequence data were deposited in the European Nucleotide Archive (ENA) under accession number PRJEB4796.

#### (ii) RAD-Seq

RAD sequencing was undertaken for five randomly selected family groups (both parents and six offspring; n = 40 individuals) from each of the four year-group subpopulations that comprise the LNS broodstock population. For each year-group, four RAD libraries were constructed; two parental libraries (five individuals each) and two offspring libraries (15 individuals each). Equimolar amounts of all four libraries were combined and run on a single Illumina Hiseq 2000 lane, giving three-fold deeper coverage of parental samples *cf*. offspring. The RAD library preparation protocol employed in this study has been fully documented elsewhere [49]. Essentially it is the methodology originally described by Baird *et al.*[10] and comprehensively detailed by Etter *et al.*[50], with minor procedural modifications. In brief, DNA was extracted using Biosprint96 DNA extraction kits (Qiagen, UK) following the manufacturers protocol and treated with RNase to remove residual RNA. DNAs were quantified by spectrophotometry (Nanodrop), quality assessed by agarose gel electrophoresis, and was finally diluted to a concentration of 50 ng/μL in 5 mmol/L Tris, pH 8.5. Each sample (1.5 μg parental DNA or 0.5 μg offspring DNA) was digested at 37°C for 45 minutes with *Sbf*I high fidelity restriction enzyme (New England Biolabs, USA; NEB) using 6U *Sbf*I per μg genomic DNA in 1× Reaction Buffer 4 (NEB) at a final concentration of c. 1 μg DNA per 50 μL reaction volume. Following heat inactivation at 65°C for 20 minutes, individual specific P1 adapters, each with a unique 5 base barcode were ligated to the *Sbf*I digested DNA. Following heat inactivation individual ligation reactions were then combined in appropriate multiplex pools / libraries (5 parental samples or 15 offspring samples each). Shearing (Covaris S2 sonication) and initial size selection (250 – 500 bp) by agarose gel separation was followed by gel purification, end repair, dA overhang addition, P2 paired-end adapter ligation, library amplification, as in the original RAD protocol [46]. A total of 150 μL of each amplified library (14 - 16 PCR cycles) was size selected (c. 300 - 550 bp) by gel electrophoresis and eluted into 20 μL EB buffer (MinElute Gel Purification Kit, Qiagen, UK.) Libraries were accurately quantified by qPCR (Kapa Library), combined as appropriate and run on an Illumina Hiseq 2000. Two of the four year-class sample sets were pair-end sequenced, the other two were single end sequenced (v3 chemistry; 100 base reads). Raw reads were processed using RTA 1.12.4.2 and Casava 1.6 (Illumina, USA) and all sequence data were deposited in the ENA under accession numbers PRJEB4783 (paired-end data) and PRJEB4785 (single-end data).

#### (iii) RNA-Seq

The sequence data used to generate the RNA-Seq SNP dataset were part of a larger ongoing study with the aim of investigating the transcriptome of Atlantic salmon fry with disparate genetic resistance to the Infectious Pancreatic Necrosis Virus (IPNV). Briefly, three families derived from Landcatch (UK) broodstock were challenged with IPNV at the Centre for Environment, Fisheries and Aquaculture Science (Cefas) in Weymouth, UK. Details on the challenge protocol have been described previously [9]. From each family, one group of fry were sampled prior to challenge and one group were sampled one day post-challenge and stored at -80°C until processing. Fish were euthanised using a non-schedule 1 method under a procedure specifically listed on the appropriate Home Office (UK) license and all experiments were performed under approval of Cefas ethical review committee and complied with the Animals Scientific Procedures Act [45].

RNA-Seq libraries each comprised of six individual homogenised whole fry (each ~0.5 g) per family per timepoint (total n = 72). Each fry was homogenised in 5 ml TRI Reagent (Sigma, USA) using a Polytron mechanical homogeniser (Kinemetica, Switzerland). The RNA was isolated from 1 ml of the homogenate, using 0.5 vol. RNA precipitation solution (1.2 mol/L sodium chloride; 0.8 mol/L sodium citrate sesquihydrate) and 0.5 vol. isopropanol. Following re-suspension in nuclease-free water, the RNA was purified using the RNeasy Mini kit (Qiagen, UK). The RNA integrity numbers from the Bioanalyzer 2100 (Agilent, USA) were all over 9.9. Thereafter, the Illumina Truseq RNA Sample Preparation kit v1 protocol was followed directly, using 4 μg of RNA per sample as starting material. Libraries were checked for quality and quantified using the Bioanalyzer 2100 (Agilent, USA), before being sequenced in barcoded pools of 12 individual fish on the Illumina Hiseq 2000 instrument (100 base paired-end sequencing, v3 chemistry) and all sequence data were deposited in the ENA under accession number ERP003968.

### SNP discovery and filtering

#### RR-Seq

All reads were aligned to the Atlantic salmon reference genome assembly (NCBI Assembly GCA_000233375.1) using BWA 0.5.9 [51], allowing up to 4 mismatches per 100 bases. Predicted allele frequencies were derived from SAMtools [52] mpileup v0.1.19 using default settings. To exclude putative PSVs, genotypes were called in the library derived from the haploid Atlantic salmon embryo using GATK UnifiedGenotyper v2.1.9 [53] and any SNP showing a heterozygous genotype (genotype quality >20) was removed (n = 133,029). Of the putative SNPs remaining, those with an allele frequency of ≤ 0.1 or a read depth of ≤ 10 (n = 172,501) were removed. Finally, SNPs occurring within known genomic repeat elements defined according to the salmonid-specific repeat-masker (http://grasp.mbb.sfu.ca/GRASPRepetitive.html) (n = 67,445) were removed leaving 99,097 candidate RR-Seq-derived SNPs.

#### RAD-Seq

All reads were aligned to the Atlantic salmon reference genome assembly (NCBI Assembly GCA_000233375.1) using BWA 0.5.9 [51], allowing up to 4 mismatches per 100 bases. Duplicated reads originating from PCR were marked using Picard and subsequently ignored. GATK UnifiedGenotyper v 2.1-9 [53] was used to detect and genotype putative SNPs, enabling the base-alignment quality (BAQ) calculation and otherwise using the default parameters. Genotypes with a quality score of >20 were retained and SNPs that demonstrated two or more mendelian errors or significant mendelian distortion (chi2 P <0.05) in any of the families (n = 344,278) were removed. The remainder were repeat-masked as above (39,839 removed) leaving 83,151 candidate RAD-Seq-derived SNPs.

#### RNA-Seq

Bowtie2 v2.1.0 alignment software [54] was used for alignment of the generated RNA-seq reads with requirements of a perfect end-to-end and gapless alignment of seed substrings of 32-mers. Each sample was aligned to the Atlantic salmon genome assembly (NCBI Assembly GCA_000233375.1). SAMtools v0.1.19 [52] was then used to identify any SNPs within the aligned sequences or between the Atlantic salmon genome assembly and the aligned sequences. SNP calls were generated with default SAMtools [52] pileup settings and standard SNP filters. Only the 426,135 transversions (which are best-suited for inclusion on the Axiom array) with a predicted MAF ≥ 0.1 were retained. These were repeat-masked as above (196,381 removed) which left 229,754 candidate RNA-Seq-derived SNPs.

All newly discovered filtered SNPs from the RR-Seq, RAD-Seq and RNA-Seq experiments were submitted to dbSNP (NCBI ss# 947429275 - 947844429) [55].

#### Publicly-available and other SNPs

A non-exhaustive list of publicly-available Atlantic salmon SNPs (n = 9,084) was created as an additional set of candidate SNPs for inclusion on the ssalar01 array. This list included all SNPs in dbSNP [55] which included the SNPs described in Lien *et al.*[3], the SNPs described in Moen *et al.*[34] and the QTL-linked SNPs described in Houston *et al.*[12]. An additional eight unpublished SNPs discovered in our laboratories were added. The flanking sequence for these SNPs was aligned to the reference genome and the SNPs were included as candidates for submission to Affymetrix if they mapped to a single unique genomic location and contained sufficient flanking sequence for probe design (30 bases of flanking sequence).

### Y-specific probes for test of genetic sex

A homology search of the rainbow trout Y-specific master sex-determining gene sdY ([45] Accession AB626896.1) identified an Atlantic salmon EST (Accession CK897399.1) comprising part of Exon 4 and the 3′UTR sequence of SRY in Atlantic salmon. Using PCR primer sets designed from both these sequences, partial sequences from the SRY gene in Atlantic salmon were obtained by direct sequencing of amplicons. Two contigs were produced (Additional file 4); a 2,149 nt fragment comprising most of exon 2 and exon 3 with intervening intron and a 1,147 nt fragment comprising exon 4 with partial upstream intron and downstream 3′ UTR sequence. Following repeat masking using the salmonid-specific repeat-masker (http://grasp.mbb.sfu.ca/GRASPRepetitive.html), a series of 87 partly overlapping, potentially Y-specific probes (Additional file 5) were designed to both DNA strands, according to Axiom non-polymorphic gender probe guidelines [56].

### Affymetrix Axiom array creation and genotyping

The candidate SNPs were provided to Affymetrix as 71-mer nucleotide sequences from the forward strand with the alleles at the target SNP highlighted at position 36. Using proprietary software, ‘p-convert’ values (representing the probability of a given SNP converting to a reliable SNP assay on the Axiom array system; see [26]) were computed for each submitted SNP sequence. Potential probes were designed for each SNP in both the forward and reverse direction, each of which is designated as ‘recommended’, ‘neutral’, or ‘not recommended’ based on p-convert values. All ‘recommended’ probes were included and ‘neutral’ probes were included if paired with a ‘recommended’ or a ‘neutral’ probe, resulting in the tiling of probes for 266,105 putative SNPs, this being 93% of the capacity of the array. To fill the remainder of the array, the following categories of putative SNPs were included: (i) putative SNPs discovered in more than one sequencing experiment with low p-convert score; (ii) SNPs mapping to two locations in the reference genome with a p-convert score over 0.6; and (iii) previously verified SNPs (from the ‘Publicly available and other’ category) with a non-zero p-convert score. In the final array, most of the SNPs are interrogated by two independent probesets, designed at the 5′ and at the 3′ of the SNP. The R package ‘SNPolisher’ is used to choose the best performing probeset for every SNP. A probeset will have one or two different probes on the array, depending on the base change (A/T and C/G SNPs require two different probes). Each probe is tiled twice on the array, which means that there are two identical independent copies of each probe spatially separated on the array to provide robustness against potential local image artifacts. During the analysis, the signal from the two probes is summarized to provide a single signal estimate for each SNP.

A test plate of 96 genomic DNA samples from Atlantic salmon of various sources was genotyped using the ssalar01 array (Additional file 2). These samples comprised 47 representative samples of Atlantic salmon distributed across all four yeargroups of the Landcatch Natural Selection (Ormsary, UK) breeding program (termed ‘Farmed Scottish’), eight Atlantic salmon originating from Aquagen (Trondheim, Norway) and eight Atlantic salmon originating from Salmobreed (Bergen, Norway) (together termed ‘Farmed Norwegian’), 24 Atlantic salmon from Br5 and Br6 SalMap families [41], three Atlantic salmon sourced from the River Dee (Scotland), two from the River Corrib (Ireland), two from the River Hopselv (Norway) and two from the River Lerez (Spain) (together termed ‘Wild’). Details of the Axiom SNP genotyping and quality-control procedures are given elsewhere [37,56]. Briefly, each SNP allele generates a hybridisation signal and the size and contrast of these signals is computed for each SNP for each individual to generate genotype clusters using the Axiom GT1 algorithm. The analysis consists of a pre-processing stage which includes image artefact reduction and an algorithm that filters out contiguous probes with unexpected intensity level, if they occur. This is followed by a quantile normalization on the two Axiom channels separately and median polish summarization to generate intensity signals for the A and B alleles. For the genotype calling, the allele-signal estimates derived in the pre-processing stage are the input values to the clustering algorithm. These signal values are transformed into the contrast-size (also called MvA) space used for clustering [56] defined in the following way: Contrast = logA – logB, and Size = (logA + logB)/2. The first stage of clustering evaluates all possible placements of two vertical boundaries (to define three genotype clusters) between data on the X axis, computing for each a posterior likelihood given the data and a Bayesian prior on cluster locations. After identifying the labeling of maximum likelihood, the prior two-dimensional Gaussian mixture model is updated in a Bayesian fashion to produce a posterior model that is used to make genotype calls; the same posterior can also be used as a prior for future clusterings. In the final stage, genotype calls are assigned by associating each sample to the closest posterior model.

The SNPs were split into categories according to their clustering performance with respect to various Axiom-generated quality-control criteria; (i) ‘polymorphic high resolution’ where the SNP passes all QC, (ii) ‘monomorphic high resolution’ where the SNP passes all QC except the presence of a minor allele in two or more samples, (iii) ‘call rate below threshold’ where genotype call rate is under 97%, (iv) ‘no minor homozygote’ where the SNP passes all QC but only two clusters are observed, (v) ‘off-target variant’ (OTV) where atypical cluster properties arise from variants in the SNP flanking region, and (vi) ‘other’ where the SNP does not fall into any of the previous categories. OTVs are reproducible and previously uncharacterized variants that interfere with genotyping a SNP and usually display substantially low hybridization intensities and are centred at zero in the contrast dimension (A - B). This could be due to a SNP in the flanking sequence of one or both Atlantic salmon paralogues. This can result in miscalling of individuals as heterozygous (AB). However, they usually sit below the heterozgous cluster on the y-axis [(A + B)/2]. Such miscalled heterozygotes can be identified using ‘OTV_Caller’ which is part of the SNPolisher (an R package available from Affymetrix). The Expectation-Maximization (EM) algorithm is used with the posterior information to identify which samples should be in the OTV cluster and which samples should remain in the AA, AB, or BB clusters. In this study, only SNPs from categories (i) and (iv) were included in further analyses. These filtered SNP data were analysed for allele frequency distribution and Mendelian inheritance using the software Plink [44].

### Linkage analysis

To map a subset of the QC-filtered SNPs to chromosomes, a sire-based linkage analysis was performed for a subset of offspring in the two ‘SalMap’ families [41] (all parents and 10 offspring per family; total n = 24) using the CriMap software [42] as modified by Xuelu Liu (Monsanto, USA). This analysis relied on the lack of male recombination in centromeric regions of the male salmonid genome, and this feature facilitated mapping of markers to linkage groups according to identical or near-identical sire-based inheritance patterns. The number of offspring per family was too small to determine marker positions within those linkage groups. Firstly, the QC-filtered SNPs which had the segregation pattern AB (sire) × AA or BB (dam) in at least one of the families were identified. Secondly, a ‘two-point’ linkage analysis was performed to determine the LOD scores between all pairs of markers in randomly selected pools of ~5,000 SNPs including anchor markers from each of the 29 pairs of Atlantic salmon chromosomes (Additional file 1: Table S2). Thirdly, the ‘autogroup’ option was used to cluster markers into linkage groups, starting with more stringent parameters and proceeding to less stringent parameters. The parameter settings for ‘autogroup’ were: Layer 1 (5, 2.0, 4, 0.9); Layer 2 (4, 1.5, 4, 0.7); Layer 3 (3, 1.0, 4, 0.6); Layer 4 (2.5, 0.5, 4, 0.3). The final layer corresponded to a LOD score of 2.5 which was necessarily lower than the typical threshold of 3.0 to include SNPs that were segregating in only one sire and were inherited without recombination (LOD ~ 2.7). For chromosomes 2 and 6, and chromosomes 22 and 23, the sire-based inheritance pattern was very similar in one of the families which resulted in conflicting linkage assignments. Therefore, those linkage groups were defined using sire-segregation of markers in the other family only.

### Identity-by-state clustering

The ability of the ‘ssalar01’ Axiom array to identify distinct genetic populations and population structure was evaluated on all the unrelated samples (as Table 3) based on pairwise IBS distance calculated using the software Plink [44]. A multidimensional scaling analysis on the *N* × *N* matrix of genome-wide IBS pairwise distances was performed and a scatterplot of the individuals based on their position on the first two dimensions was created.

### Availability of supporting data

The sequencing data from this study have been deposited in the European Nucleotide Archive (ENA) http://www.ebi.ac.uk/ena/ under accession numbers PRJEB4796, PRJEB4783, PRJEB4785 and ERP003968, and the SNP details have been submitted to dbSNP https://www.ncbi.nlm.nih.gov/SNP/ under NCBI ss# 947429275 - 947844429. Other supporting data are available as additional files.

## Competing interests

Two commercial organisations (Landcatch Natural Selection Ltd and Affymetrix Ltd) were involved in the development of the Atlantic salmon SNP array and preparation of the manuscript. Among the authors, AT and AH work for Landcatch Natural Selection Ltd, AleD and FB work for Affymetrix Ltd and FB holds shares in Affymetrix Ltd. The array will be made available for purchase as a catalogue array from Affymetrix Ltd from March 2014. This project was funded by a Technology Strategy Board grant promoting industry-academia collaboration.

## Authors’ contributions

Conceived, designed and managed the experiments: RDH, JBT, RT, SCB, ALA, JEB, DJP, FB, AET, KG, AH. Performed the laboratory experiments: JBT, NRL, AliD, DJP. Analyzed the data: RDH, TC, MB, AleD. Wrote the manuscript: RDH. All authors read and approved the final manuscript.

## Supplementary Material

Additional file 1: Table S1 Summary of the number of SNPs discovered in single and multiple sequencing experiments for candidate SNPs included on the array and for final QC-filtered SNPs. **Table S2.** Details of the microsatellite markers used for the linkage analysis to anchor SNP markers to chromosomes. Click here for file

Additional file 2 **Details of the 96 samples used for testing the ‘ssalar01’ array.** List of genotyped samples including the population, phenotypic sex (if known), sire (if genotyped), dam (if genotyped), and the SNP discovery experiment which the sample was also used (if applicable). Click here for file

Additional file 3 List of the SNP markers mapped to chromosomes/linkage groups using sire-based linkage mapping. Click here for file

Additional file 4 Partial sequence of the Atlantic salmon sdY gene used for Y-specific probe design. Click here for file

Additional file 5 Sequence details for the Y-specific probes placed on the array to provide a molecular genetic test for sex. Click here for file
