# Supplementary material for: Development and validation of a high density SNP genotyping array for Atlantic salmon (Salmo salar)
Source: BMC Genomics. 2014 Feb 6;15:90. doi: 10.1186/1471-2164-15-90 (PMC3923896; doi:10.1186/1471-2164-15-90)
Supplement: Additional file 1: Table S1 — Summary of the number of SNPs discovered in single and multiple sequencing experiments for candidate SNPs included on the array and for final QC-filtered SNPs. Table S2. Details of the microsatellite markers used for the linkage analysis to anchor SNP markers to chromosomes. [file 1471-2164-15-90-S1.docx]

Table S1 - Summary of the number of SNPs discovered in single and multiple sequencing experiments

| **SNP discovery category** | | | | **Number of candidate SNPs on array** | **Number of final QC-filtered SNPs** |
| --- | --- | --- | --- | --- | --- |
| **RR-Seq** | **RAD-Seq** | **RNA-Seq** | **Other *** |  | |
| X |  |  |  | 71,088 | 51,549 |
|  | X |  |  | 51,937 | 25,497 |
|  |  | X |  | 154,910 | 48,844 |
|  |  |  | X | 4,447 | 3,383 |
| X | X |  |  | 1,351 | 1,005 |
| X |  | X |  | 1,188 | 993 |
| X |  |  | X | 145 | 131 |
|  | X | X |  | 808 | 505 |
|  | X |  | X | 72 | 59 |
|  |  | X | X | 20 | 19 |
| X | X |  | X | 2 | 2 |
|  | X | X | X | 2 | 2 |
| X |  | X | X | 1 | 1 |
| **Total:** |  |  |  | **286,021** | **132,033** |

* ‘Other’ consists mainly of publicly-available SNPs from dbSNP (see ‘Methods’)

Table S2 - Details of the anchor markers used for the linkage analysis

| **Atlantic salmon linkage group** | **Atlantic salmon chromosome** | **Marker name** | **Marker type** | **GenBank accession number (if known)** |
| --- | --- | --- | --- | --- |
| 1 | 2 | Omy11/1INRA  OmyFGT8/1TUF  Oneµ18  Ssa202  Ssa406UOS  Ssa-A14/1  Ssa-A15/1  Str4/1INRA |  | U56718.1  U43695.1  AJ402723.1 |
| 2 | 10 | Ogo8  Oneµ5  Ssa-A13  Str-A3/2 |  | AF009780 |
| 3 | 14 | Ssa0014ECIG  Ssa0033ECIG  Ssa0169bECIG |  | 119096977  119096998  119097160 |
| 4 | 6 | Omy27/1INRA  OmyFGT1TUF  OmyRGT30/2TUF  Ssa171  Ssa-A12 |  | U43693.1 |
| 5 | 13 | Sfo23  Ssa420UOS  SSsp2201  Str-A5  Str-A8/1 |  | AJ402737.1  AY081807.1 |
| 6 | 12 | Omy11/2INRA  Omy21INRA  Omy27DU  OmyFGT25TUF  OmyRGT35TUF  SSsp2210 |  | AB087604.1  AY081808.1 |
| 7 | 24 | Ocl9  SSsp2215  SSsp2216 |  | AF028698  AY081810.1  AY081811.1 |
| 8 | 15 | Omy27/2INRA  Omy301UoG  Oneµ9  Ssa197  Ssa401UOS  Ssa-A60  Str-A3/1  Str-A8/2  Str-A8/3 |  | U56709.1  U43694.1  AJ402718.1 |
| 9 | 11 | Ssa132  Ssa408UOS  Ssa413UOS  SSspG7 |  | U58901.1  AJ402725.1  AJ402730.1  AY081813.2 |
| 10 | 9 | MST541INRA  Ogo2/2  OmyFGT21TUF  OmyRGT30/1TUF  Oneµ7  Ssa412UOS  Ssa45/2micUOS  Ssa-A33  Ssa-A34/2  Str85INRA |  | U56707.1  AJ402729.1  SRX000001 |
| 11 | 3 | Oki2  OmyRGT32TUF  Ssa417UOS |  | AF055428  AB087602.1  AJ402734.1 |
| 12 | 5 | Ocl2  Omy272/2UoG  OmyFGT8/2TUF  Ssa-A14/2  Ssa-A15/2  Str15INRA  Str4/2INRA  Str-A9/1 |  | AF028699 |
| 13 | 19 | MC4R  Ssa289  Ssa407UOS  Ssa422UOS |  | AJ402724.1  AJ402739.1 |
| 14 | 21 | Str-A22/1  Str-A22/2/1 |  |  |
| 15 | 27 | Ssa0122aECIG |  | 119097105 |
| 16 | 18 | OmyRGT55TUF  Ssa416UOS  Str-A12/1  Str-A9/2 |  | AB031201.1  AJ402733.1 |
| 17 | 1 | Ogo3  OmyRGT34TUF  Ssa14  Ssa410UOS  Str-A12/2  Str-A22/2/2 |  | AF009795  AB031199.1  AJ402727.1 |
| 18 | 23 | OmyFGT16TUF  Ssa85  SSsp1605 |  | U43692.1  AY081812.1 |
| 19 | 8 | Ssa0136ECIG  Ssa0158ECIG |  | 119097123  119097148 |
| 20 | 25 | MEP-2* |  |  |
| 21 | 26 | IDDH-2*  OmyRGT44TUF  Ssa-A45/2/1 |  | AB087611.1 |
| 22 | 17 | Ssa12  Ssa402/2UOS  Ssa404UOS |  | U58900  AJ402719  AJ402721.1 |
| 23 | 16 | Ssa402/1UOS  Ssa403UOS |  | AJ402719  AJ402720.1 |
| 24 | 7 | Omy14INRA  Ssa418/1UOS  Ssa-A34/1 |  | AJ402735 |
| 25 | 20 | Ocl1/1  Oki10  Omy23INRA  OmyFGT14TUF  OmyFGT34TUF  Ssa421UOS  Ssa-A10 |  | AF028694  AF055435  AJ402738.1 |
| 28 | 4 | Ssa405UOS |  | AJ402722.1 |
| 30 | 29 | Ogo4  Ssa-A11 |  | AF009796 |
| 31 | 28 | AAT-4*  SSA224 |  | AF019168.1 |
| 32 | 22 | Ssa419UOS  Ssa-A45/1 |  | AJ402736.1 |
